# Imeglimin confers protection against intestinal ischemia/reperfusion injury in rats through AMPK-dependent inhibition of NF-κB/NLRP3 inflammasome signaling and mitigation of oxidative stress and apoptosis

**DOI:** 10.1007/s10787-026-02338-8

**Published:** 2026-07-24

**Authors:** Walaa Yehia Abdelzaher, Hanaa Mohamed Khalaf, Rabeh Khairy Saleh, Nehal Refaat Raouf, Youssra Magdy Hassan, Maggi Mofeed Ayad, Rania Rady Fadl

**Affiliations:** 1 Department of Medical Pharmacology, Faculty of Medicine, Minia University, Minya, 61519 Egypt; 2 Faculty of Oral and Dental Medicine, Lotus University, Minya, 61768 Egypt; 3 Department of Medical Pharmacology, Faculty of Medicine, Minia National University, Minya, Egypt; 4 Department of Pathology, Faculty of Medicine, Minia University, Minya, 61519 Egypt; 5 Department of Public Health and Preventive Medicine, Faculty of Medicine, Minia University, Minya, 61519 Egypt; 6 Department of Anatomy, Faculty of Medicine, Minia University, Minya, 61519 Egypt; 7 Occupational Medicine and Environmental Health, Faculty of Medicine, Minia University, Minya, 61519 Egypt

**Keywords:** Imeglimin, Intestinal ischemia reperfusion, AMPK, NLRP3, NF-κB

## Abstract

**Background and aim:**

Intestinal ischemia/reperfusion (I/R) injury is a critical condition characterized by oxidative stress, inflammation, and apoptosis, leading to significant tissue damage. Imeglimin (IMEG), a novel antidiabetic agent, has recently shown cytoprotective effects through modulation of mitochondrial function, oxidative stress, and inflammatory pathways. We aimed to investigate the potential protective effects of IMEG against II/R injury and to explore the underlying mechanisms involved.

**Methodology:**

Thirty-two adult male Wistar albino rats were divided into four groups; sham group, IMEG group, intestinal I/R group, IMEG + Intestinal I/R group. Oxidative stress markers [malondialdehyde (MDA), reduced glutathione (GSH)], and histopathological changes were assessed. Biochemical analyses included measurement of phosphorylated AMP-activated protein kinase (p-AMPK), NOD-like receptor protein 3 (NLRP3) and caspase-3. Also, gene expression of interleukin (IL)-1β, caspase-1, apoptotic Bcl-2-associated protein x (BAX) and anti-apoptotic B-cell leukemia/lymphoma 2 protein (Bcl-2) were measured. Nuclear factor-κB (NF-κB) immuno expression was estimated.

**Results:**

MDA, NLRP3, caspase-3 levels, IL-1β, caspase-1, Bax gene expression, and NF-κB immunohistochemical expression were all significantly elevated in the intestinal I/R group while GSH, p-AMPK levels and Bcl-2 gene expression were significantly decreased. Every metric indicated a notable improvement with IMEG.

**Conclusion:**

IMEG exerts a protective effect against intestinal I/R injury through its antioxidant, anti-inflammatory, and anti-apoptotic properties (Fig. 1).

**Supplementary Information:**

The online version contains supplementary material available at https://doi.org/10.1007/s10787-026-02338-8.

## Introduction

Ischemia–reperfusion (I/R) injury is considered a major clinical challenge. Evidence from both experimental and clinical settings suggests that ischemia alone does not cause the most severe damage; rather, the restoration of blood flow triggers a surge in reactive oxygen species (ROS), which are primarily responsible for cellular injury and subsequent tissue damage (Wu et al. 2018). Intestinal I/R injury is a prevalent and critical pathological condition (Kalogeris et al. 2017), commonly resulting from factors such as major trauma, burns, shock, intestinal volvulus, mesenteric thromboembolism, and small intestine transplantation (Wen et al. 2019). This type of injury is life-threatening and extends beyond the intestine, often triggering a systemic inflammatory response syndrome along with multiple organ dysfunction syndrome, both of which contribute significantly to morbidity and mortality (Li et al. 2022).

The pathophysiology of intestinal I/R injury is highly complex. The intestinal mucosal response to I/R can generally be categorized into two stages. The first stage occurs during ischemia and is characterized by tissue hypoxia and initial organ damage. The second stage occurs during reperfusion, where the reintroduction of oxygen leads to excessive ROS generation, resulting in oxidative stress, impairment of the intestinal mucosal barrier, enhanced vascular permeability, bacterial translocation, and activation of inflammatory mediators and apoptotic pathways (Li et al. 2022).

Nuclear factor-kappa B (NF-κB), a key transcription factor that regulates inflammatory responses, becomes activated during I/R. This activation promotes the production of multiple pro-inflammatory mediators, including interleukin-1beta (IL-1β), IL-6, and tumor necrosis factor-alpha (TNF-α) (Sizemore et al. 2002; Gachpazan et al. 2021). AMP-activated protein kinase (AMPK) is widely recognized as a key energy-sensing enzyme that plays vital roles at both the cellular and systemic levels. Evidence from several studies suggests that AMPK can mitigate the damage caused by I/R by suppressing NF-κB–driven inflammatory pathways (Chen et al. 2018). Therefore, AMPK is increasingly being considered a promising therapeutic target for inflammation associated with I/R injury.

Imeglimin (IMEG) is a novel oral antidiabetic agent that has gained attention for its unique mechanism of action targeting mitochondrial function. Unlike traditional hypoglycemic drugs, IMEG improves glucose homeostasis by enhancing insulin secretion, increasing insulin sensitivity, and reducing hepatic glucose production. Importantly, it exerts protective effects on mitochondrial bioenergetics, reducing the overproduction of ROS and improving cellular energy balance (Singh et al. 2023).

Recent experimental studies have emphasized the potential pleiotropic effects of IMEG beyond glycemic control. Due to its ability to modulate oxidative stress and preserve mitochondrial integrity, IMEG has been investigated in conditions associated with mitochondrial dysfunction and inflammation. Several studies have demonstrated its role in reducing oxidative stress, limiting inflammatory responses, and protecting tissues from cellular injury (Kaji et al. 2024; Ohguro et al. 2025).

**Fig. 1:**
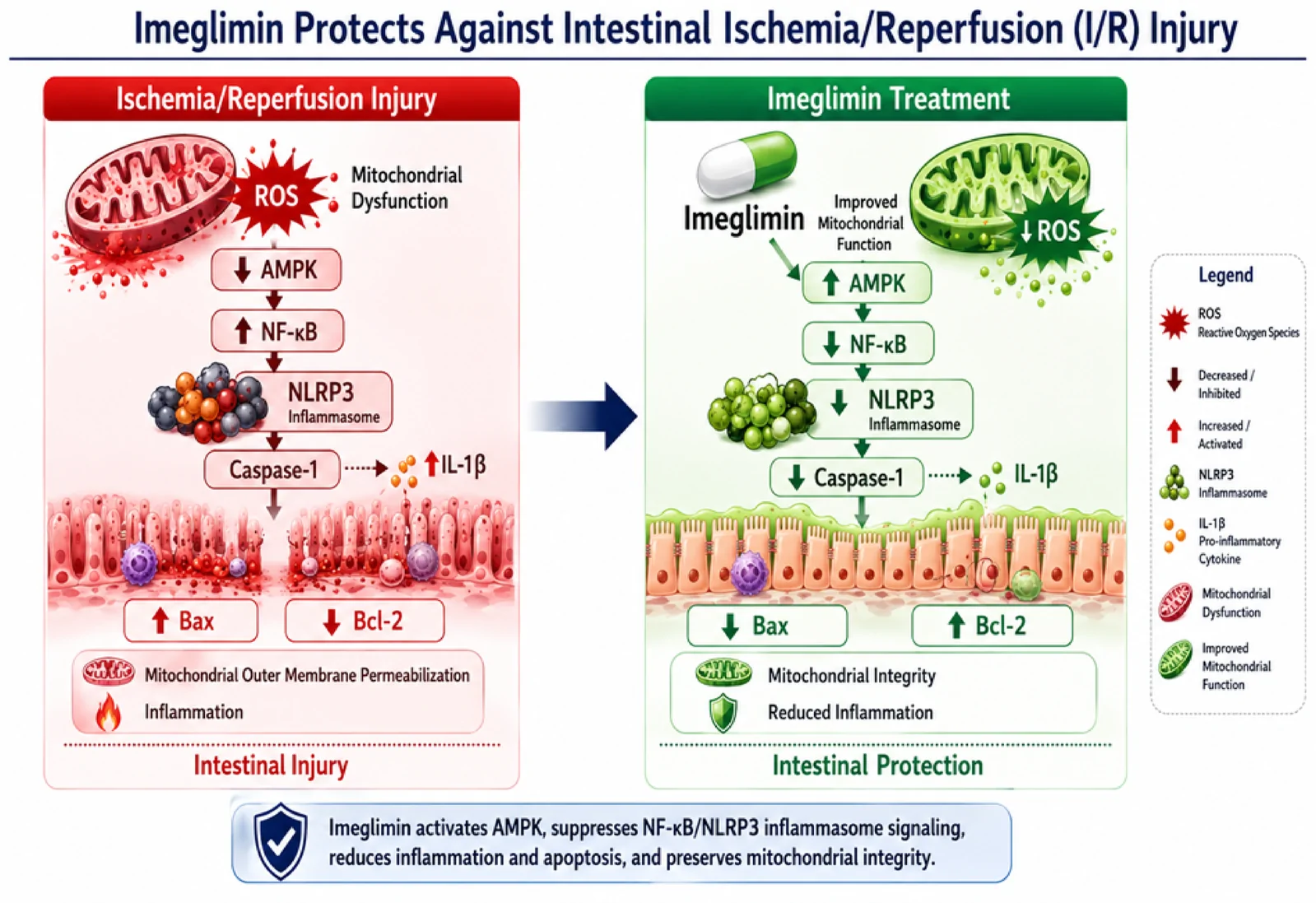
Graphic abstract of Imeglimin protective effects against intestinal I/R injury. IMEG activates p-AMPK, suppress NF-κB/NLRP3 inflammasome signaling, reduces inflammation and apoptosis and preserves mitochondrial integrity

Given that intestinal I/R injury is strongly associated with excessive ROS production, mitochondrial dysfunction, and inflammatory activation, IMEG may offer a promising therapeutic approach. Its antioxidant, mitochondria-targeting properties, anti-inflammatory and anti-apoptotic effects suggest a potential role in attenuating intestinal damage and improving outcomes in I/R-related conditions (Fig. 1).

## Materials and methods

### Ethics

Animal handling, treatment, and scarification were conducted in accordance with the NIH Guide for the Care and Use of Laboratory Animals. Ethical approval was obtained from the Institutional Ethical Committee, Faculty of Medicine, Minia University, Egypt (Approval No: 1701/09/2025).

### Chemicals and drugs

IMEG was purchased from Karman Pharmaceutical Industries, Beniswif, Egypt. All other chemicals were of the highest analytical grade and were purchased from commercial sources.

### Animals and experimental design

The current study included thirty-two adult male Wistar albino (180–220 g). The Animal Research Center in Giza, Egypt, provided the animals. Rats were housed in a standard housing facility (three rats per cage), fed chow and given tap water, and allowed to acclimate for a week. Rats were divided into four groups at random, each with eight rats. After the acclimatization period, rats were randomly allocated to the experimental groups using a lottery method. Each animal was assigned an identification number, and numbered slips were randomly drawn to determine group allocation. To minimize potential confounding effects of baseline body weight differences, animals were distributed to achieve comparable body weight ranges among the groups.

1. Sham group: With the exception of laparotomy and superior mesenteric artery exploration, rats were kept under heat lamps for the same period of ischemia and a single oral dose of 1 mL of 0.1% aqueous CMC solution was administered as a vehicle via gavage.
2. IMEG group: rats received imeglimin 150 mg/kg/day, orally for 7 days (Muramatsu et al. 2026).
3. Intestinal I/R group: rats received a single oral dose of 1 mL of 0.1% aqueous CMC solution via gavage, followed by laparotomy with occlusion of the superior mesenteric artery for 30 min and subsequent 90 min of reperfusion (Kamel et al. 2020; Refaie et al. 2024).
4. IMEG + Intestinal I/R group: rats received imeglimin 150 mg/kg/day orally for 7 days before Intestinal I/R.

*The doses and duration were selected according to our pilot study and references mentioned previously.*

### Surgical procedures

Following a single intraperitoneal dose of urethane anesthesia (1 g/kg), the abdomen was disinfected with Betadine, and a midline abdominal incision was performed; and the superior mesenteric artery was constricted for 30 min using microvascular bulldog clamps. After that, the clamps were removed to perform intestinal reperfusion for 90 min, and a heating pad was used to maintain the rats’ body temperature at 37 degrees Celsius. As control groups, the sham-operated rats underwent comparable surgical procedures without superior mesenteric artery blockage (Kamel et al. 2020; Refaie et al. 2025).

### Blood and tissue samples

At the conclusion of the trial, rats were scarificed by an intraperitoneal injection of urethane (1 g/kg). The rats’ abdominal aortas were used to draw blood samples. After that, it was centrifuged for 15 min at 4000 × g using a Janetzki T30 centrifuge (Germany). The obtained sera were then stored at − 80 °C for use in biochemical analysis. Saline was used to wash the intestinal tissue, and the separated portion was stored at − 80 °C for biochemical examination. For histological analysis, another portion of intestinal tissue was placed in 10% formalin.

### Biochemical determination

#### Estimation of intestinal oxidative stress parameters

Potassium phosphate buffer (10 mM pH 7.4) was used to homogenize intestinal tissue. Intestinal tissue was homogenized in a 1:5 w/v ratio. The homogenates were centrifuged at 4000 × g for 10 min at 4 °C. In order to estimate biochemical parameters, the resultant supernatant was aspirated and collected.

The level of malondialdehyde (MDA), a biomarker of lipid peroxidation, was measured using the technique outlined by Buege and Aust (1978).

We bought reduced glutathione (GSH) from Biodiagnostic in Egypt. The manufacturer’s recommendations were followed while quantifying it.

#### Estimation of inflammatory and apoptotic parameters

ELISA kits (MyBioSource, Inc., San Diego, USA, and Catalogue No. MBS 164089, MBS2033695, and MBS018987, respectively) were used to assess intestinal AMPK, NLRP3, and caspase-3. According to the manufacturer’s instructions, all were quantified.

### Real-time reverse-transcription polymerase chain reaction (RT-PCR) of intestinal caspase-1, IL-1β, BAX and Bcl-2 gene expression

In intestinal tissue, caspase-1, interleukin-1beta (IL-1β), the apoptotic Bcl-2-associated protein x (BAX) and anti-apoptotic B-cell leukemia/lymphoma 2 protein (Bcl-2) genes were assessed by relative quantification using the RT-PCR method. Following the manufacturer’s recommendations, isolation of total RNA from the homogenized intestinal samples was conducted by the use of the Ribozol RNA extraction reagent (Amresco, Solon, USA). Using a RevertAidTM First Strand copy DNA (cDNA) Synthesis kit, cDNAs were created (Fermentas, Life Sciences). For the reverse transcription of 2 μg of total RNA into cDNA, a utilization of M-MuLV reverse transcriptase and an RNase inhibitor was conducted. In the Real-Time PCR Detection System, RT-PCR was carried out with 5 ng of cDNA per reaction through the use of 25 μL of SYBR Green QPCR Mix (Solis BioDyne) containing 20 μM of specific primers. GAPDH (glyceraldehyde- 3-phosphate dehydrogenase) served as the endogenous control gene for normalization in SYBR Green- based relative quantification. The primers employed in this study were (Table 1). The formula 2 (ΔΔCt) was used to determine the relative expression level of each gene (VanGuilder et al. 2008). They were normalized to the control samples, which were assigned a value of 1, and the results of each experimental group were expressed graphically as relative expression levels compared to the control.

**Table 1:** The primers of caspase-1, IL-1β, BAX and Bcl-2

The parameter	The primer
Caspase-1	5′-CACAGCTCTGGAGATGGTGA-3′ and reverse, 5′-TGTGAGTGGTGCTGTCAGAG-3′
IL-1β	5′-GCAACTGTTCCTGAACTCAACT-3′ and reverse, 5′-ATCTTTTGGGGTCCGTCAACT-3′
BAX	5′-TATATGGCCCCAGC ATGCGA-3′, and reverse, 5′-GGGCAGGTTTGTCG ACCTCA-3′
Bcl-2	5′-TATATGGCCCCAGC ATGCGA-3′, and reverse, 5′-GGGCAGGTTTGTCG ACCTCA-3′

IL-1β, Interleukin-1beta; BAX, Apoptotic Bcl-2-associated protein x; Bcl-2, Anti-apoptotic B-cell leukemia/lymphoma 2 protein

### Histopathology and immunohistochemical study

#### Histopathology staining and scoring

For light microscopy analysis, the small intestinal tissues were prepared, embedded in paraffin wax, and preserved in 10% neutral buffered formalin. Then, the tissues in paraffin wax were cut into sections 4 μm thick, put on slides, and stained with hematoxylin and eosin (H&E) (Bancroft and Layton 2013).

The Chiu-Park scoring system, which offers a score that reflects gradations of ischemia injury from no injury to full loss of villi and transmural infarction, was used to evaluate the degree of small intestine injury. Grade 0: normal mucosa; Grade 1: formation of a sub-epithelial space at the tips of the villi; Grade 2: more extended sub-epithelial space at the tips of the villi, development of Gruenhagen’s space at the tips of the villi; Grade 3: massive epithelial lifting down the sides of the villi, villus necrosis; Grade 4: villi are denuded of epithelial layer; Grade 5: loss of villi, mucosal ulceration and necrosis with invasion of the muscularis propria; Grade 6: Crypt layer destruction; Grade 7: Transmucosal infarction and Grade 8: Transmural infarction. The median scores were used to compare between groups (Park et al. 1990; Wilken et al. 2025).

#### Immunohistochemical staining and scoring

The small intestine mucosa’s NF-kB protein expression was assessed using immunohistochemistry. In short, the sections were first deparaffinized in xylene and rehydrated in a descending ethanol series. After de-waxing and rehydration, antigen was retrieved by microwave for 15 min and endogenous peroxidase activity was blocked by 3% H2O2 in methanol at room temperature for 20 min. The sections were probed with specific polyclonal rabbit anti-rat NF-kB serum for 12 h at 4° C. Negative control was incubated with normal rabbit serum under the same conditions. After incubation with the primary antibody, the slides were washed by PBS and incubated with the respective secondary antibody and 0.1% diaminobenzidine substrate. Ten fields were randomly selected (400 × magnification), and the results were quantified as 0 = no staining (negative), 1 = staining < 25% of area, 2 = staining 25–50% of area and 3 = strong staining > 50% of area (Wen et al. 2014; Su et al. 2019).

Histopathological and immunohistochemical evaluations were performed by an independent pathologist who was blinded to the experimental groups during tissue examination and scoring.

### Statistics

#### Sample size calculation

G power program version (3.1.9) was used to calculate sample size for this study with priory analysis. F tests were used to detect the difference between 4 independent means regarding the intestinal GSH as the primary outcome. Based on mean and standard error of mean (SEM) of intestinal GSH in the study (Refaai et al. 2025) the calculated effect size was 0.8 (large effect size), alpha error was 0.05 and power of 0.95 was used. The total sample size was 32 and grouped into 4 groups each having 8 rats.

#### Statistical analysis

Tukey’s post hoc multiple comparison tests were employed after one-way ANOVA. Means ± SEM and 95% confidence intervals were used to present the results. GraphPad Prism (version 5) and SPSS version 27 were utilized for the analysis and figures. For significance, a *P*-value of less than 0.05 was used.

## Results

### Effect of IMEG on intestinal oxidative stress parameters in intestinal I/R in rats

The intestinal I/R group showed a significant increase in intestinal MDA with a significant decrease in intestinal GSH levels, when compared to sham and IMEG groups. On the other hand, IMEG + intestinal I/R treated group showed a significant improvement in oxidative stress parameters when compared to the intestinal I/R group (Table 2).

**Table 2:** Effect of IMEG on oxidative stress parameters in intestinal I/R in rats

Groups	Intestinal MDA (nmol/g tissue(	95% CI	Intestinal GSH (nmol/g tissue(	95% CI
Sham	44.64 ± 3.51	36.3–52.9	127.40 ± 5.07	115.3–139.4
IMEG	50.78 ± 3.02	43.6–57.9	128.90 ± 5.51	115.9–141.9
Intestinal I/R	112.10 ± 5.83^a/b^	98.3–125.9	46.35 ± 2.37^a/b^	40.7–51.9
IMEG + Intestinal I/R	59.90 ± 3.67^c^	51.2–68.6	113.90 ± 7.62^c^	95.8–131.9

One-way ANOVA test, F: 55.043

Data are expressed as mean ± SEM. (n = 8 rats/group (.Values were considered significantly different when *P* < 0.05

IMEG, Imeglimin; Intestinal I/R, Intestinal ischemia reperfusion; MDA, Malondialdehyde; GSH, Reduced glutathione

^a^Significant difference from sham group

^b^Significant difference from IMEG group

^c^Significant difference from intestinal I/R group

### Effect of IMEG on intestinal inflammatory parameters in intestinal I/R in rats

**Fig. 2:**
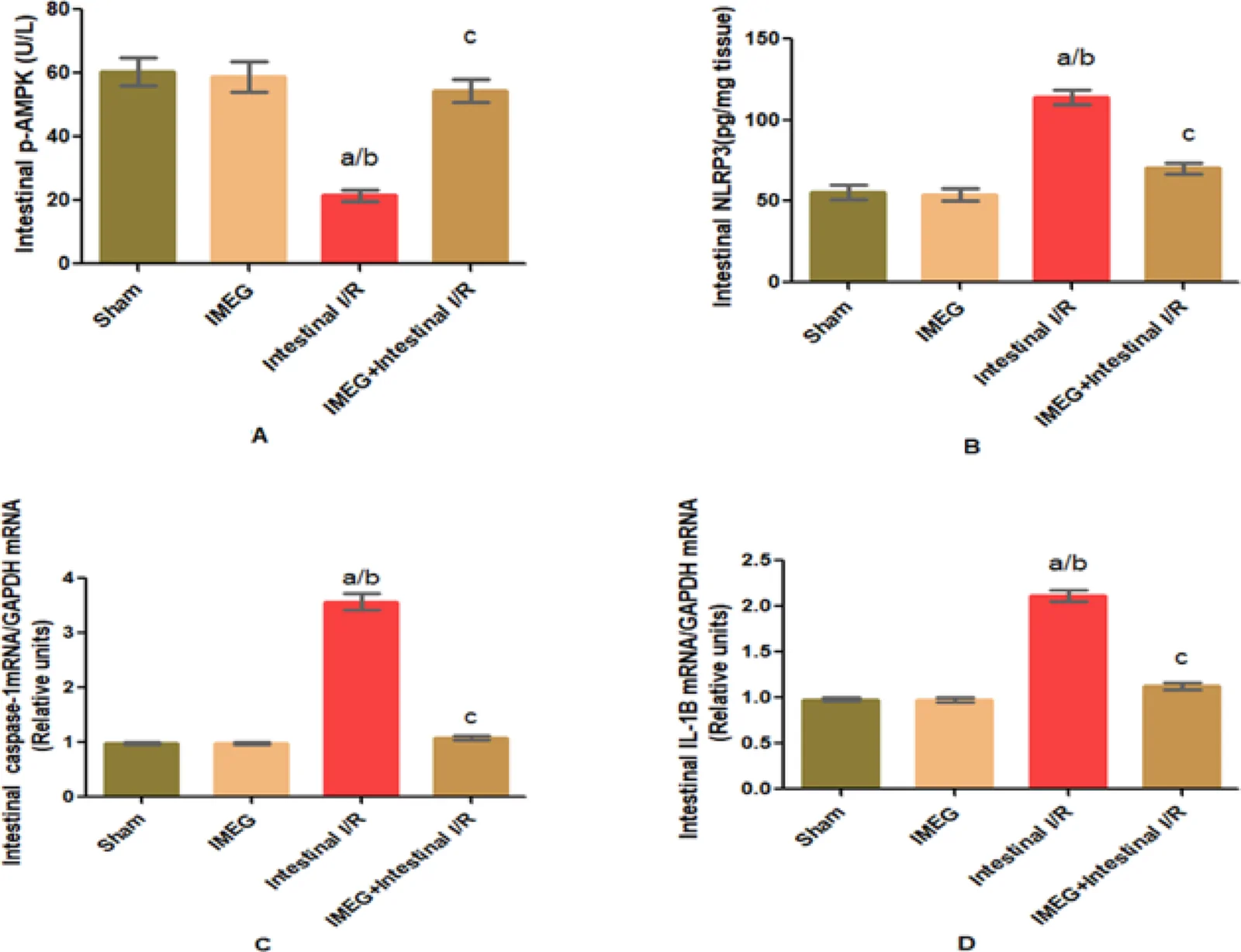
Effect of IMEG on intestinal inflammatory parameters in intestinal I/R in rats. Data are expressed as mean ± SEM. (n = 8 rats/group). Intestinal I/R significantly decreased p-AMPK expression and increased NLRP3, caspase-1 and IL-1β levels compared with the sham group. IMEG treatment significantly restored p-AMPK expression and attenuated the elevations of NLRP3, caspase-1 and IL-1β induced by intestinal I/R. Values were considered significantly different when *P* < 0.05. ^a^Significant difference from sham group, ^b^Significant difference from IMEG group, ^c^Significant difference from intestinal I/R group. IMEG, Imeglimin; Intestinal I/R, Intestinal ischemia reperfusion; p-AMPK, Phosphorylated AMP-activated protein kinase; NLRP3, NOD-like receptor protein 3; IL-1β, Interleukin-1beta. p-AMPK (SHAM group mean: 60.5, 95 CI: 50.4–70.6, IMEG group mean: 58.9, 95% CI 47.4–70.5, intestinal I/R group mean: 21.5, 95% CI 17.5–25.6, IMEG + intestinal I/R group mean: 54.5, 95% CI 45.9–63.1). NLRP3 (SHAM group mean: 55.9, 95 CI: 45.3–66.3, IMEG group mean: 54.3, 95% CI 45.2–63.4, intestinal I/R group mean: 114.2, 95% CI 102.8–125.5, IMEG + intestinal I/R group mean: 70.3, 95% CI 62.1–78.6). Caspase-1 (SHAM group mean: 0.98, 95 CI: 0.94–1.03, IMEG group mean: 0.98, 95% CI 0.93–1.04, intestinal I/R group mean: 3.57, 95% CI 3.21–3.93, IMEG + intestinal I/R group mean: 1.07, 95% CI 0.99–1.2). IL-1β (SHAM group mean: 0.98, 95 CI: 0.93–1.02, IMEG group mean: 0.98, 95% CI 0.92–1.04, intestinal I/R group mean: 2.1, 95% CI 1.97–2.25, IMEG + intestinal I/R group mean: 1.13, 95% CI 1.03–1.22)

The intestinal I/R group showed a significant increase in intestinal NLRP3 level, caspase-1 and IL-1β gene expressions, with a significant decrease in intestinal p-AMPK level when compared to sham and IMEG groups. Meanwhile, IMEG + intestinal I/R treated group showed a significant decrease in intestinal NLRP3 level, caspase-1 and IL-1β gene expressions, with a significant increase in intestinal p-AMPK level when compared to the intestinal I/R group (Fig. 2).

### Effect of IMEG on intestinal apoptotic parameters in intestinal I/R in rats

**Fig. 3:**
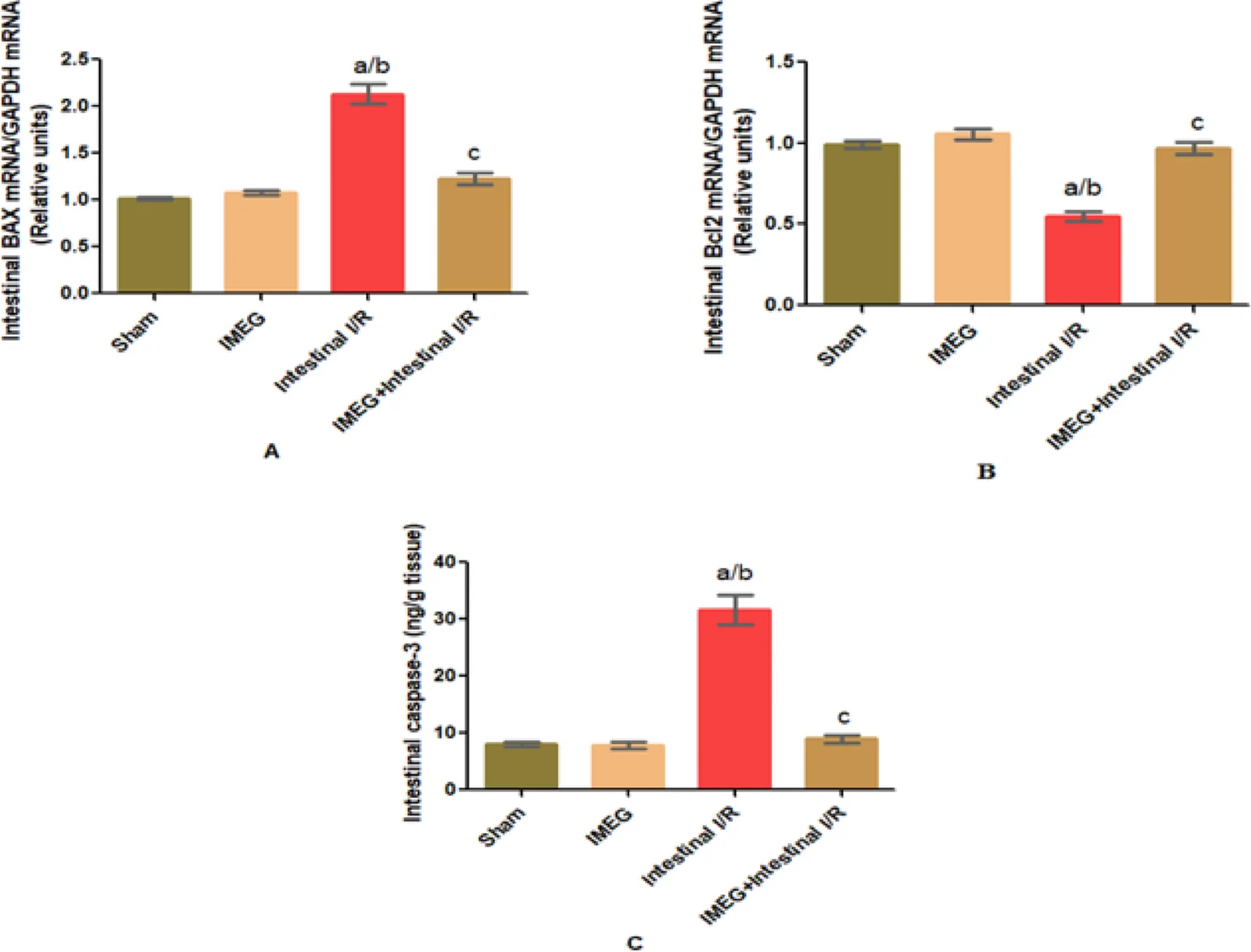
Effect of IMEG on intestinal inflammatory parameters in intestinal I/R in rats. Data are expressed as mean ± SEM. (n = 8 rats/group). Intestinal I/R significantly increased the expression of the pro-apoptotic markers BAX and caspase-3 and decreased the expression of the anti-apoptotic marker Bcl-2 compared with the sham group, indicating enhanced apoptotic activity. IMEG treatment significantly attenuated these alterations, as evidenced by reduced BAX and caspase-3 expressions and increased Bcl-2 expression compared with the intestinal I/R group. Values were considered significantly different when *P* < 0.05. ^a^Significant difference from sham group, ^b^Significant difference from IMEG group, ^c^Significant difference from intestinal I/R group. IMEG, Imeglimin; Intestinal I/R, Intestinal ischemia reperfusion; BAX, Apoptotic Bcl-2-associated protein x; Bcl-2, Anti-apoptotic B-cell leukemia/lymphoma 2 protein. BAX (SHAM group mean 1.01, 95 CI: 0.99–1.05, IMEG group mean: 1.08, 95% CI 1.01–1.14, intestinal I/R group mean: 2.1, 95% CI 1.87–2.37, IMEG + intestinal I/R group mean: 1.22, 95% CI 1.08–1.38). Bcl-2: (SHAM group mean: 0.99, 95 CI: 0.94–1.04, IMEG group mean: 1.05, 95% CI 0.98–1.12, intestinal I/R group mean: 0.55, 95% CI 0.48–0.61, IMEG + intestinal I/R group mean: 0.97, 95% CI 0.88–1.06). Caspase-3: (SHAM group mean: 8.1, 95 CI: 7.1–9.1, IMEG group mean: 7.9, 95% CI 6.6–9.1, intestinal I/R group mean: 31.7, 95% CI 25.6–37.7, IMEG + intestinal I/R group mean: 8.9, 95% CI 7.2–10.7)

Figure 3 shows a significant increase in intestinal BAX gene expression and caspase-3 level, with a significant decrease in intestinal Bcl-2 gene expression in the intestinal I/R group when compared to the sham and IMEG groups. IMEG + intestinal I/R treated group showed a significant decrease in intestinal BAX gene expression and caspase-3 level, with a significant increase in intestinal Bcl-2 gene expression, when compared to the intestinal I/R group.

### Histological results

**Fig. 4:**
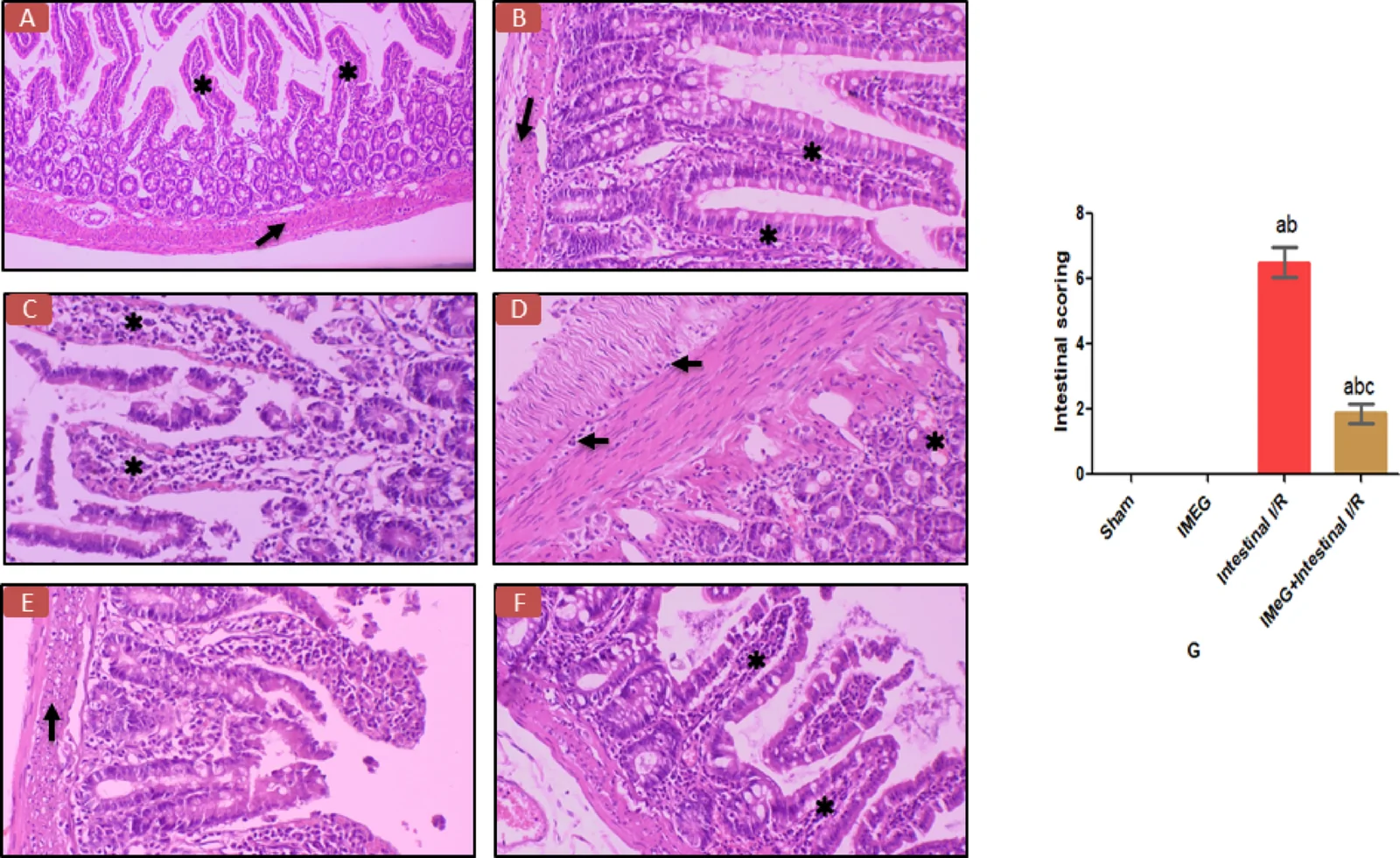
H&E stain of the rat small intestinal groups: (**A**) Sham group, the intestinal mucosa (score 0) for the intestinal villi (asterisks) and muscle layer (arrow) (H&E, × 100). (**B**) IMEG group, showed no significant injury (score 0) in intestinal villi (asterisks) and muscle layer (arrow) (H&E, × 200). (**C**) Intestinal I/R group, the mucosa showed necrosis of the intestinal villi (score 3) (asterisks) (H&E, × 200). (**D**) Intestinal I/R group, the intestinal mucosa showed inflammatory cellular infiltration reaching the musculosa (arrows) and congestion (asterisk) (H&E, × 400). (**E**) Intestinal I/R group, the intestinal mucosa showed necrosis of the intestinal villi (asterisks) and transmural injury (score 8) (arrow) (H&E, × 200). (**F**) IMEG + Intestinal I/R group, shows no significant pathological changes in the intestinal villi (asterisks) (H&E, × 200). Intestinal I/R induced severe intestinal injury characterized by villous necrosis, inflammatory cell infiltration, congestion, and transmural damage, resulting in significantly elevated histopathological scores. IMEG treatment markedly attenuated these pathological changes and improved intestinal tissue architecture compared with the intestinal I/R group. Data are expressed as mean ± SEM. (n = 8 rats/group).Values were considered significantly different when *P* < 0.05. ^a^Significant difference from sham group, ^b^Significant difference from IMEG group, ^c^Significant difference from intestinal I/R group. IMEG, Imeglimin; Intestinal I/R, Intestinal ischemia reperfusion. Intestinal scoring: (intestinal I/R group mean: 6.5, 95% CI 25.6–37.7, IMEG + intestinal I/R group mean: 1.9, 95% CI 7.2–10.7) independent t test *P* value < 0.0001, Cohen’s d effect size 4.213 (95% CI 2.363–6.020)

H&E stain of intestinal tissue of both sham and IMG groups showed no significant injury (score 0) in intestinal villi and muscle layer. IIR group mucosa showed necrosis of the intestinal villi (score 3), inflammatory cellular infiltration reaching the musculosa, and congestion with transmural injury (score 8). IMG + IIR group showed no significant pathological changes in the intestinal villi (Fig. 4).

**Fig. 5:**
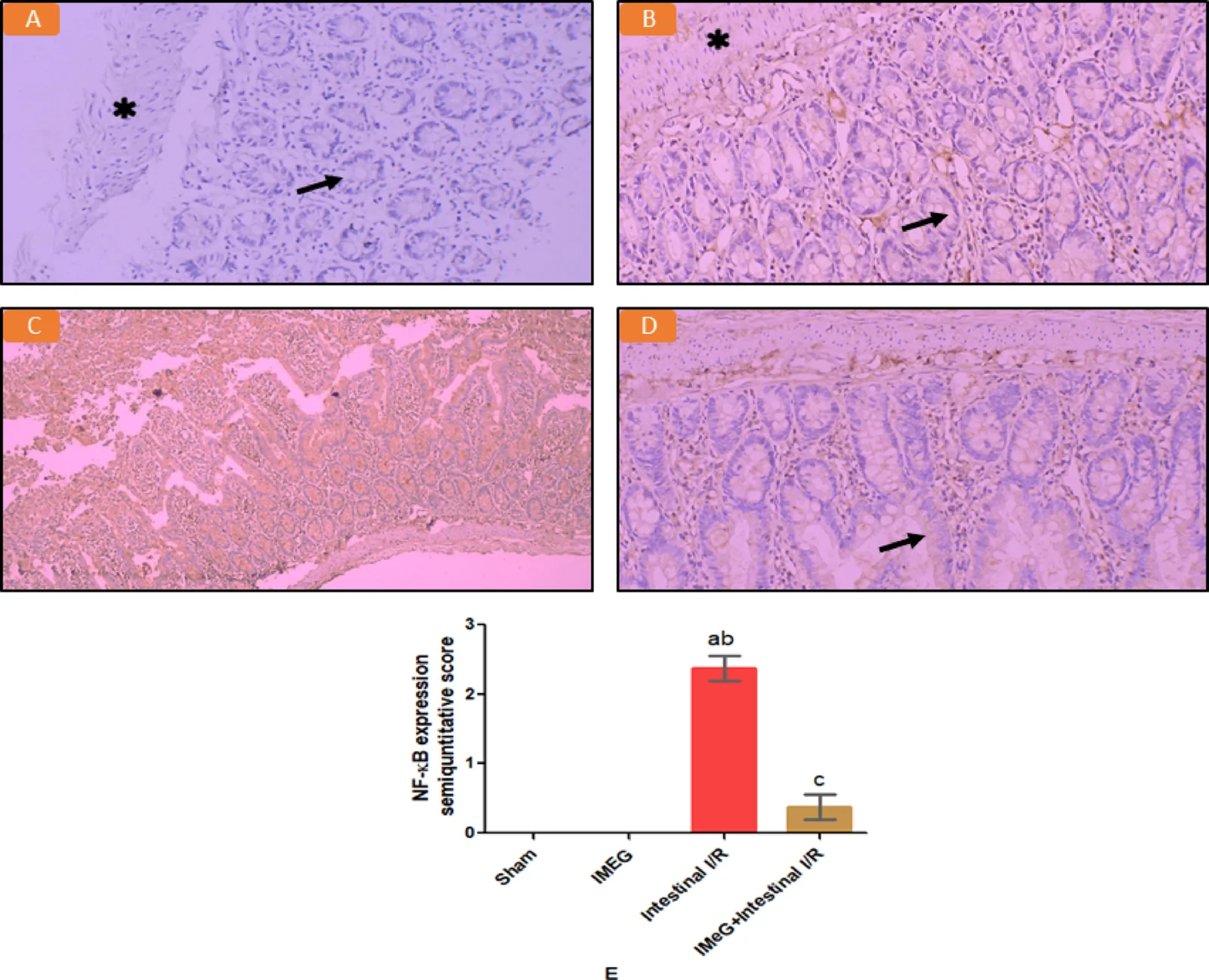
IHC expression of NF-κB in small intestine groups: (**A**) NF-κB expression in sham group, the picture demonstrates negative expression in the small intestine including mucosa (arrow) and musculosa (asterisk) (IHC, × 200). (**B**) NF-κB expression in IMEG group, the picture demonstrates no expression in the small intestinal mucosa (arrow) and musculosa asterisk) (IHC, × 400). (**C**) NF-κB expression in intestinal I/R group, the picture demonstrates diffuse expression in the intestinal mucosa and injured intestinal villi (IHC, × 200). (**D**) NF-κB expression in IMEG + Intestinal I/R group, the picture demonstrates scattered focal expression in the intestinal villus (arrow) (IHC, × 400). Minimal or absent NF-κB immunoreactivity was observed in the sham and IMEG groups. Intestinal I/R markedly increased NF-κB expression in the intestinal mucosa and damaged villi, whereas IMEG treatment significantly reduced NF-κB immunostaining compared with the intestinal I/R group, indicating attenuation of the inflammatory response. Data are expressed as mean ± SEM. (n = 8 rats/group). Values were considered significantly different when *P* < 0.05. ^a^Significant difference from sham group, ^b^Significant difference from IMEG group, ^c^Significant difference from intestinal I/R group. Semi-quantitative scoring confirmed the visual observations shown in the representative photomicrographs. IMEG, Imeglimin; Intestinal I/R, Intestinal ischemia reperfusion; NF-kB, Nuclear factor-kappa B. NF-κB: intestinal I/R group mean 2.38, IMEG + intestinal I/R group 0.38, *P* value < 0.0001, Cohen’s d 3.86 (95% CI 2.12–5.56)

Immunohistochemical expression of NF-κB in the small intestine showed negative expression in both sham and IMG groups. IIR demonstrated diffuse expression in the intestinal mucosa and injured intestinal villi. IMG + IIR group showed scattered focal expression in the intestinal villus (Fig. 5).

## Discussion

Intestinal I/R injury remain a major clinical challenge characterized by excessive oxidative stress, inflammatory activation, and apoptotic cell death. The present study demonstrates that IMEG significantly attenuates intestinal I/R-induced injury, as evidenced by reduced oxidative stress, suppression of inflammatory mediators, inhibition of apoptosis, and preservation of intestinal histological architecture. These protective effects appear to be associated with activation of AMPK and subsequent inhibition of the NF-κB/NLRP3 signaling pathway.

Oxidative stress is a key initiating event in intestinal I/R injury. Reperfusion following ischemia results in excessive production of ROS, leading to lipid peroxidation, depletion of endogenous antioxidants, cellular dysfunction, and tissue injury (Sahna et al. 2005; Wu et al. 2020). Consistent with these observations, the present study demonstrated increased MDA levels and decreased GSH content following intestinal I/R. IMEG significantly restored redox balance, indicating potent antioxidant activity. These findings agree with previous reports demonstrating that IMEG improves mitochondrial function and reduces oxidative stress in several experimental models (Kaji et al. 2024; Refaie et al. 2025; Bagul et al. 2025).

A major finding of the present study is that the protective effect of IMEG appears to involve coordinated modulation of the AMPK/NF-κB/NLRP3 signaling axis. Intestinal I/R markedly suppressed p-AMPK activation while enhancing NF-κB expression and NLRP3 inflammasome activity, resulting in increased production of IL-1β and activation of downstream inflammatory and apoptotic cascades. IMEG treatment reversed these alterations, suggesting that p-AMPK activation may represent an upstream regulatory mechanism that limits NF-κB-mediated inflammatory signaling and subsequent NLRP3 inflammasome activation. Since oxidative stress generated during reperfusion serves as a potent trigger for both NF-κB and NLRP3 activation, suppression of this pathway may account for the observed anti-inflammatory effects of IMEG (Hu et al. 2021; Luo et al. 2022; Ghafouri-Fard et al. 2022).

The observed effects of IMEG are comparable to those reported for several established cytoprotective agents in I/R injury. Metformin, a well-characterized AMPK activator, attenuates I/R-induced tissue injury through suppression of NLRP3 inflammasome activation, reduction of inflammatory cytokine release, and inhibition of apoptosis (Zhang et al. 2020). Similarly, resveratrol exerts protective effects by enhancing antioxidant defenses and suppressing NF-κB-dependent inflammatory signaling (Mokni et al. 2013; Zhang et al. 2026). Melatonin has also been shown to alleviate I/R-induced damage by reducing oxidative stress, preserving mitochondrial function, and inhibiting NLRP3 inflammasome activation (Ozacmak et al. 2005; Yang et al. 2020). Therefore, the protective profile observed with IMEG appears to share common mechanistic pathways with these established cytoprotective agents while potentially providing additional benefits through improvement of mitochondrial bioenergetics (Hyodo et al. 2025; Li et al. 2025).

The histopathological findings further support the biochemical and molecular results. Intestinal tissues exposed to I/R exhibited severe mucosal disruption, epithelial degeneration, villous damage, and inflammatory cell infiltration; these findings are consistent with previous experimental studies (Higa et al. 2007; Li et al. 2009; Gordeeva et al. 2017). In contrast, IMEG treatment markedly preserved intestinal architecture and reduced inflammatory infiltration, providing morphological evidence for its protective effect against I/R-induced tissue injury.

Apoptosis represents a crucial mechanism contributing to intestinal damage following I/R. In agreement with previous reports, intestinal I/R significantly increased BAX and caspase-3 expression while reducing Bcl-2 levels, indicating activation of the mitochondrial apoptotic pathway (Kamel et al. 2020; Afolabi et al. 2022; Zhang et al. 2022). IMEG reversed these alterations, suggesting a potent anti-apoptotic effect. These findings are supported by previous studies showing that IMEG reduces caspase-3 activation, limits cytochrome c release, and preserves mitochondrial integrity in different experimental models (Fauzi et al. 2022; Sanada et al. 2022; Kato et al. 2024). The anti-apoptotic effect observed in the present study is likely secondary to attenuation of oxidative stress and inflammatory signaling together with improvement of mitochondrial function.

Collectively, the present findings suggest that IMEG protects against intestinal I/R injury through coordinated attenuation of oxidative stress, inflammation, and apoptosis, possibly via activation of p-AMPK and inhibition of NF-κB/NLRP3 signaling. While the 150 mg/kg dose of IMEG was effective in the current rat model, direct translation of this dose to clinical practice requires caution due to species-specific differences in pharmacokinetics and drug metabolism. Therefore, the present study should be viewed as providing mechanistic and proof-of-concept evidence, while further dose-scaling and clinical investigations are needed to establish the optimal therapeutic regimen in humans.

## Conclusion

The present study demonstrated that IMEG significantly attenuated intestinal I/R-induced injury, as evidenced by reduced oxidative stress, inflammation, and apoptosis, together with improved histopathological outcomes. These protective effects were associated with increased p-AMPK expression and suppression of NF-κB/NLRP3 inflammasome signaling. Although the current findings suggest a potential involvement of the AMPK/NF-κB/NLRP3 axis in the protective actions of IMEG, further mechanistic studies using pharmacological or genetic approaches are required to establish causality. Collectively, these results support the therapeutic potential of IMEG as a promising candidate for the management of intestinal I/R injury and warrant further preclinical and clinical investigation.

## Limitations of the study

Several limitations of the present study should be acknowledged. First, although IMEG treatment showed significant biochemical and molecular effects, the study was designed as a short-term experimental model, which limits evaluation of long-term outcomes and sustained therapeutic efficacy. Second, the study lacks functional assessments of intestinal recovery, such as motility or barrier function tests, which could have provided additional insight into the physiological relevance of the observed histological and molecular changes. Third, as previously noted, the current experimental design does not establish a direct causal relationship between p-AMPK activation and downstream NF-κB/NLRP3 inhibition, since no pharmacological or genetic modulation of AMPK was performed. Fourth, the study was conducted exclusively in male rats, preventing evaluation of sex-related differences. Finally, the acute rat intestinal ischemia/reperfusion model does not fully reflect the complexity of human disease, and interspecies differences may limit direct clinical translation. Further studies addressing these limitations are warranted to strengthen mechanistic understanding and translational relevance.

## Supplementary Information

Below is the link to the electronic supplementary material.


Supplementary Material 1


## Data Availability

No datasets were generated or analysed during the current study.
