# Supplementary material for: Imeglimin confers protection against intestinal ischemia/reperfusion injury in rats through AMPK-dependent inhibition of NF-κB/NLRP3 inflammasome signaling and mitigation of oxidative stress and apoptosis
Source: Inflammopharmacology. 2026 Jul 24;34(8):5705–16. doi: 10.1007/s10787-026-02338-8 (PMC13525062; doi:10.1007/s10787-026-02338-8)
Supplement: Supplementary file 1 [file 10787_2026_2338_MOESM1_ESM.docx]

**Table S1: Effect of IMEG on different parameters in intestinal ischemia reperfusion model in rats**

| **Groups** | **Intestinal MDA**  **(nmol/g tissue(** | **Intestinal GSH**  **(nmol/g tissue(** | **Intestinal p-AMPK**  **(U/L)** | **Intestinal NLRP3**  **(pg/mg tissue)** | **Intestinal caspase-3**  **(ng/g tissue)** |
| --- | --- | --- | --- | --- | --- |
| **Sham** | **44.64±** **3.51** | **127.40±** **5.07** | **60.54±4.27** | **55.81±4.43** | **8.09±0.42** |
| **IMEG100** | **40.62±2.64** | **127.1±5.59** | **58.08±3.34** | **53.04±2.27** | **8.42±0.41** |
| **IMEG150** | **50.78±** **3.02** | **128.90 ± 5.51** | **58.92±4.88** | **54.28±3.86** | **7.87±0.53** |
| **IMEG200** | **46.71±2.06** | **133.1±2.64** | **61.68±3.53** | **56.79±3.60** | **7.78±0.43** |
| **Intestinal I/R** | **112.10±** **5.83^a/b^** | **46.35 ±** **2.37^a/b^** | **21.54±1.69 ^a/b^** | **114.2±4.79 ^a/b^** | **31.65±2.56 ^a/b^** |
| **IMEG100+ Intestinal I/R** | **67.57±4.12****^a/b/c^** | **94.39±4.19 ^a/b/c^** | **41.82±2.08^a/b/c^** | **87.39±2.18 ^a/b/c^** | **14.33±0.95^a/b/c^** |
| **IMEG150+ Intestinal I/R** | **59.90±** **3.67^c^** | **113.90 ±** **7.62^c^** | **54.54±3.65^c^** | **70.34±3.48^c^** | **8.96±0.74^c^** |
| **IMEG200+ Intestinal I/R** | **56.02±3.53^c^** | **130.1±7.21^c^** | **62.84±4.47^c^** | **69.02±5.35^c^** | **10.75±0.99^c^** |

Data are expressed as mean ± SEM. (n = 8 rats/group (.Values were considered significantly different when P<0.05. ^a^Significant difference from sham group, ^b^significant difference from IMEG group, ^c^significant difference from Intestinal I/R group. (IMEG= imeglimin; Intestinal I/R= intestinal ischemia reperfusion; MDA= malondialdehyde; GSH=reduced glutathione; p-AMPK= phosphorylated AMP-activated protein kinase; NLRP3=NOD-like receptor protein 3).
